# A Hidden Tumor: Unraveling a Gastrointestinal Stromal Tumor in a 44-Year-Old Male Patient With Epigastric Pain

**DOI:** 10.7759/cureus.78953

**Published:** 2025-02-13

**Authors:** Nika Harutyunyan

**Affiliations:** 1 Department of Medicine, University of California Los Angeles (UCLA) Health, Los Angeles, USA

**Keywords:** extraintestinal gist, gastric tumor, gastrointestinal stromal tumor (gist), t3n0 classification, unexplained abdominal pain

## Abstract

This case report discusses a 44-year-old male who presented with recurrent epigastric pain after starting Ozempic for weight management, which persisted despite switching to Jardiance. His symptoms evolved into diarrhea and fever while vacationing in Mexico, with notable episodes of bloody stools and greasy, floaty stools upon return home. Despite normal laboratory tests and negative stool pathogen results, a CT scan revealed a mass in the gastric antrum. Subsequent endoscopy and fine-needle aspiration confirmed a gastrointestinal stromal tumor (GIST), classified as T3N0. The patient underwent laparoscopic distal gastrectomy with Billroth II gastrojejunostomy. This case underscores the challenge of diagnosing GISTs due to nonspecific gastrointestinal symptoms, emphasizing the necessity for thorough evaluation in persistent cases. The favorable prognosis associated with T3N0 classification highlights the importance of early identification and surgical intervention. Monitoring for recurrence and considering adjuvant therapy are essential in managing such tumors. This case illustrates the value of multidisciplinary care in addressing complex gastrointestinal conditions.

## Introduction

Diagnosing gastrointestinal stromal tumors (GISTs) becomes very challenging with nonspecific gastrointestinal symptoms. Semaglutide, a glucagon-like peptide 1 (GLP-1) receptor agonist, is commonly prescribed for glycemic control in Type 2 diabetes but has also gained widespread use in weight management due to its effects on appetite suppression and gastric motility [1]. However, gastrointestinal side effects, including nausea, vomiting, and abdominal pain, are well-documented, with up to 30% of patients reporting such symptoms [2]. Empagliflozin (Jardiance) is a selective sodium-glucose co-transporter 2 (SGLT2) inhibitor. Although SGLT2 inhibitors generally have a more favorable gastrointestinal side effect profile, recent studies have noted rare cases of abdominal pain associated with these agents, particularly in patients with preexisting gastrointestinal sensitivity [3].

This report describes the case of a middle-aged patient who presented with a several-month history of recurrent epigastric pain, which began shortly after initiating semaglutide (Ozempic) for weight management. Upon subsequently switching to Jardiance, the patient continued to experience similar crampy epigastric discomfort. Apart from this, he also had a travel history to Mexico. These made the diagnosis very challenging. After different investigations, finally, fine-needle aspiration (FNA) confirmed a diagnosis of GIST mixed spindle-cell and epithelioid type classified as cT3N0.

## Case presentation

A 44-year-old male patient with a past medical history of obesity and well-controlled asthma, presented with a several-month history of recurrent epigastric pain, which began shortly after initiating semaglutide (Ozempic) for weight management. While vacationing in Mexico 2.5 months earlier, he developed diarrhea and fever, reporting four to five loose stool movements daily, which worsened upon returning home. Notably, he had one episode of blood in his stool, prompting further evaluation. After experiencing significant discomfort, the patient discontinued Ozempic and subsequently initiated Jardiance. However, the patient continued to experience similar crampy epigastric discomfort, prompting him to discontinue both medications. Despite the cessation of both medications, he reported persistent, intermittent crampy epigastric pain. Over the next two months, his diarrhea continued, characterized by floating, greasy stools with white rice-like particles. He noted a sensation of fullness after meals and occasional nausea but denied significant weight loss.

The review of systems highlighted gastrointestinal issues, including crampy abdominal pain, diarrhea, and blood in his stool. He reported experiencing a fever during his trip but no night sweats or unexplained weight loss. The patient's vitals were within normal limits with blood pressure at 143/93 mmHg, heart rate at 92 beats per minute, temperature at 98.6 degrees Fahrenheit, and oxygen saturation at 96%. Physical examination revealed a stable patient with mild epigastric tenderness but no palpable abdominal masses.

**Figure 1:**
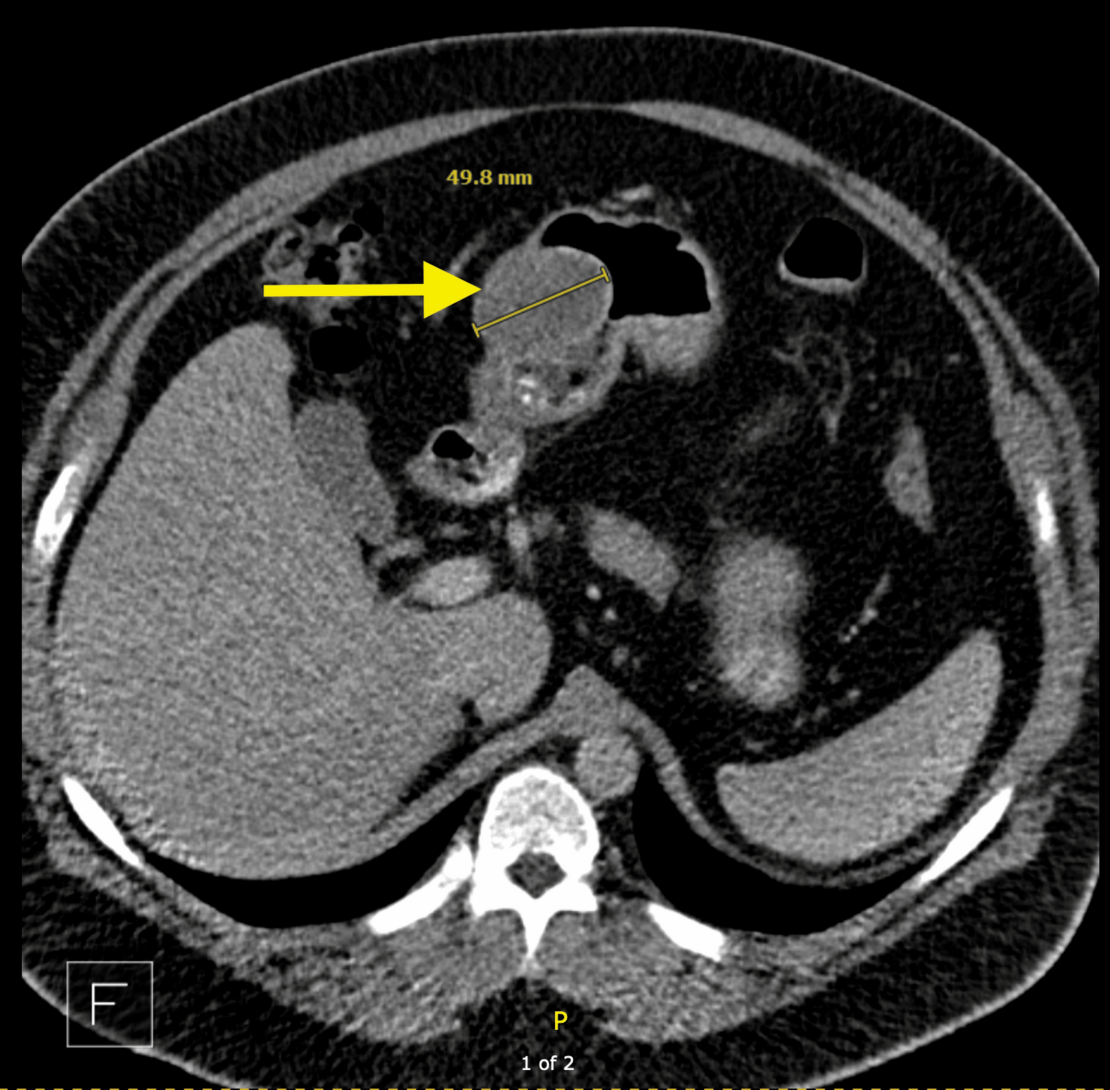
CT showing a likely submucosal mass (arrow) with smooth margins, involving the anterior gastric antrum

Laboratory tests showed slight elevations in alkaline phosphatase and lipase, while a complete blood count and comprehensive metabolic panel were normal (Table 1). Stool tests for pathogens were negative. A CT scan of the abdomen revealed a mass in the gastric antrum (Figure 1). 

**Table 1:** Relevant laboratory values * data obtained from a visit to a primary care physician

Date	Test	Patient Value (U/L)	Normal Range (U/L)
At time of presentation	Alkaline Phosphatase	135	37-113
Prior to presentation*	Alkaline Phosphatase	67	37-113
At time of presentation	Lipase	82	13-69

**Figure 2:**
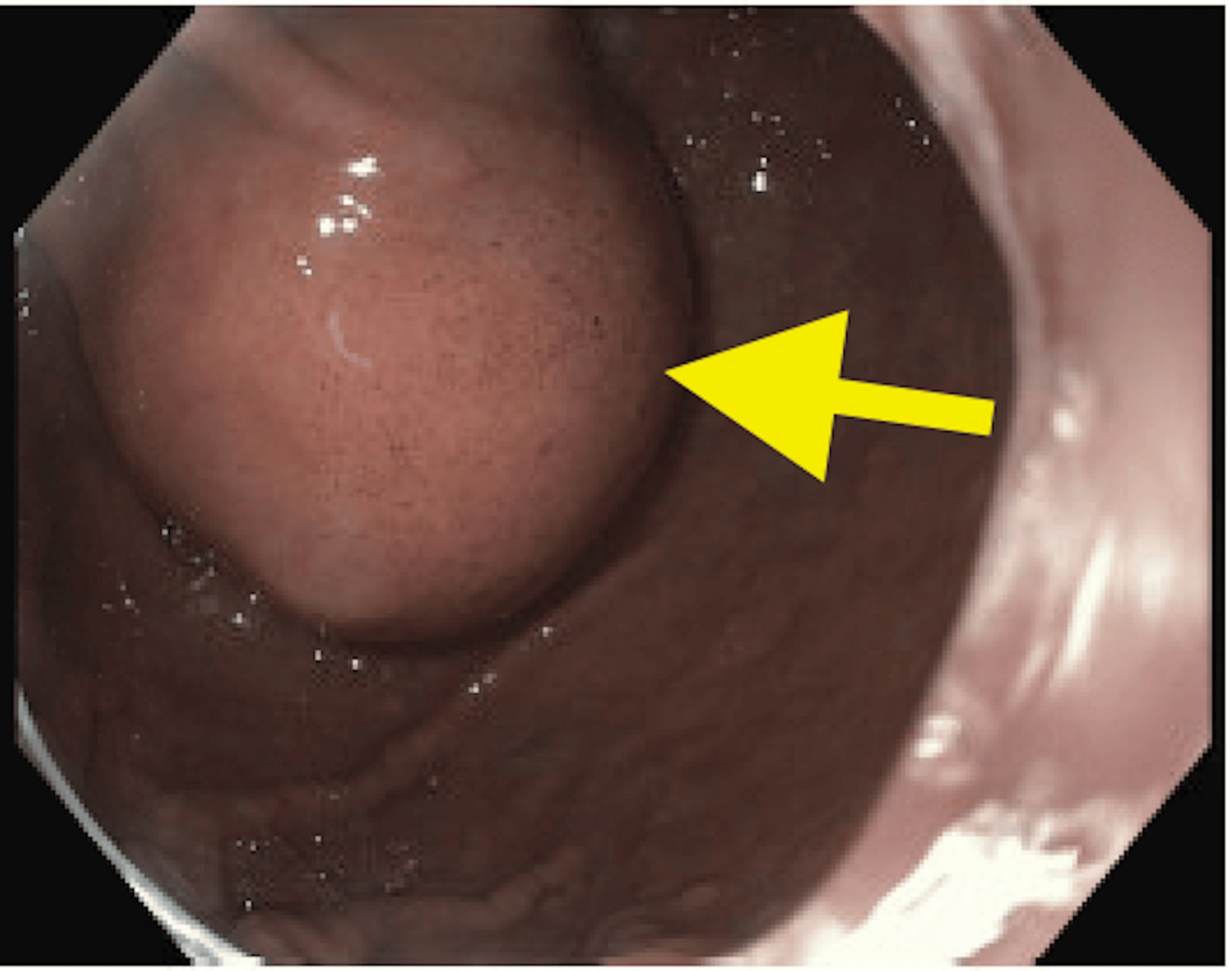
Endoscopic ultrasound showing antral mass (arrow)

Subsequently, the patient underwent upper endoscopy with endoscopic ultrasound (EUS) (Figure 2), and fine-needle aspiration (FNA), confirming a diagnosis of GIST, mixed spindle-cell and epithelioid type, measuring 5.9 cm in maximal diameter, classified as cT3N0. He then underwent laparoscopic distal gastrectomy with Billroth II gastrojejunostomy. He was then further managed by Gastroenterology.

## Discussion

GISTs are rare neoplasms arising from interstitial cells of Cajal or precursor cells in the gastrointestinal tract, making early diagnosis challenging due to nonspecific symptoms [4]. This patient's symptoms could have easily been attributed to gastrointestinal infections or medication side effects, highlighting the need for thorough investigation when symptoms persist.

Imaging studies like CT scans assist in diagnosing GISTs, confirmed through endoscopic techniques. The cT3N0 classification indicates localized disease without regional lymph node involvement, suggesting a favorable prognosis [5]. Surgical resection remains the primary treatment for localized GISTs, with laparoscopic methods increasingly preferred for better recovery outcomes.

Postoperatively, monitoring for recurrence and considering adjuvant therapy is essential, especially for larger tumors or those with high mitotic rates. GISTs may involve mutations in the c-KIT gene, which can guide targeted therapies for unresectable or metastatic disease [6].

## Conclusions

This case underscores the importance of a comprehensive evaluation in patients with unexplained gastrointestinal symptoms, particularly when medications might influence the clinical picture. The patient's symptoms, drug history, and travel history raised suspicion of an underlying pathology but the diagnosis was challenging. It is with the help of CT, endoscopy, and FNA investigations that the diagnosis of a gastric GIST was reached. Early identification and surgical intervention are critical due to the potential for malignancy and recurrence. The successful surgical outcome illustrates the value of multidisciplinary care in managing complex cases involving gastroenterology, surgery, and oncology.
